# Chicago Medical Society

**Published:** 1878-05

**Authors:** 


### Society Reports.

### CHICAGO MEDICAL SOCIETY.

Annual meeting, Monday, April 1st, 1878.

President Dr. E. INGALS in the chair; about 40 members present.

The reports of the secretary and treasurer were read.

The election of officers resulted in the unanimous re-election of Dr. E. Ingals as President; Dr. H. M. Lyman as Vice-President; Dr. D. W. Graham, as Secretary, and Dr. Chas. W. Earle as Treasurer.

The propositions submitted by the committee at the last meeting were then taken up, discussed, and adopted by a large majority.

A committee of five, Dr. Lyman, chairman, was appointed to select a central place for the next meeting.

*Maternal Impressions and Mothers' Marks*, was the title of a paper read by Dr. ROSWELL PARK.

The writer began by calling attention to the antiquity and prevalence of the popular notion that the mental condition of a woman *enceinte* can be transmitted and translated into, or perpetuated by abnormalities in the development of the fœtus, and asking whether there was any theoretical or substantial basis for such belief. As a popular belief it was unhesitatingly received by the more ignorant of the laity of the present day even, and he desired to discuss the subject from the standpoint of sound anatomical and physiological data, and thus give the *exposé* to a fallacy.

To this end he traced the blood current of both maternal and fœtal organizations, showing that at no time, from the inception of the developmental process, till its completion, was there the slightest direct vascular communication between the two; and further than this, that no corpuscle or particle of germinal matter which could bear any impress, or give any peculiar direction to future growth, could pass through the membranes which the vascular or villous walls interposed. He claimed that the fœtus got its nutritious supply in a crude gaseous or albuminous condition, by osmotic processes, and worked it up *pro re nata.*

While he allowed that all growth depended upon such supply, and that its proper regulation might be, and to a great extent was, under the control of the nervous system—and therefore more or less under that of the mind—of the mother, he insisted that the influence of any derangement of control, or of supply, could not be pre-determined, or estimated in terms of departure from the normal standard. In confirmation of these statements he appealed to the known tendency in cases of abnormality to revert to more embryonic types of growth—to interesting pathological features of these cases, and to the accepted teachings of comparative embryology.

Having disposed of the possibility of influences of this kind through the blood or nutritive supply, he considered the possibility of the transmission of such influences by other means, and quoted Whittaker, Meadows, Küss, Hermann, and others, to the effect that the placenta contained neither nerve nor lymphatic, and that the cord contained neither these, capillaries nor vasa vasorum; and that those who claimed otherwise were the ones who had not investigated for themselves.

He thought one was forced to the conclusion that the ovum and the spermatozöon contained between them all that was to differentiate the particular individual from others of the race, and that it was more truly scientific to acknowledge our total ignorance of the mysteries of heredity than to try to account for them, as toö many would do; and he would impose the burden of proof upon those who held a contrary opinion.

The ridiculous features of the subject were then touched upon, such as the intrinsic absurdity, not to say stupidity, of ascribing to accidents or impressions during the later months of pregnancy peculiarities which must have had their origin almost consentaneously with that of the embryonic mass, and before the mother was aware of her condition. A number of vices of conformation that must have their starting point thus early were mentioned—as well as many of the various pathological conditions of uterus, placenta, membranes, or ovum, which must certainly lead to some abnormality in the offspring, such as a double monster arising from a double "primitive trace."

The writer then referred to the subjective features of the case, the trying ordeal of gestation, how the mind of one mother was agitated by various and conflicting emotions and anxieties, while that of another never seemed disturbed, and denied that the percentage of marked or deformed children born to those like the former, exceeded that of similarly marked babes born to those like the latter. But, to carry the matter to its legitimate extreme, he asked if anything analagous to the matter of maternal impressions had ever been noticed among animals, and could get no answer in the affirmative. He quoted the investigations of John Hunter and Dr. Fisher (of New York State), who always inquired of patients approaching confinement (altogether more than 3,200 cases), concerning their hopes and fears relative to the subject in hand, and who never found any correspondence between apprehensions and results, nor anything which could in any way tend to substantiate the general idea.

Dr. Park closed by referring to the number of recorded cases which would seem to place the possibility of the subject of the essay beyond the pale of doubt, and said that, although many could be discarded at once as unscientific and worthless, a few were vouched for by men whose names might almost bespeak conviction, and that the most difficult matter was to decide how to receive such cases, how to accord them their proper weight in the argument. He thought that some of them might have happened as pure coincidences, than which stranger ones have occurred. Respecting the remainder, he declared we ought to suspend our judgment. The facts of such cases are so enshrouded in uncertainty, and testimony of interested parties so liable to error, that it was the only just course to pursue. This was not urged in any iconoclastic spirit—but how much better and more straightforward such a course. "Granted that strange vices of conformation do occur, we must look (on above grounds) to something deeper and beyond mere desires, caprices or mental impressions, for their explanation."

Regular meeting, Monday, April 15, 1878, at Grand Pacific Hotel.

President Dr. E. INGALS in the chair—fifty members present.

*Purpura Hemorrhagica.* Dr. N. S. DAVIS read a report of five cases of this disease, which fell under his observation during twenty years of practice in this city.

Four of the five patients were male children, and one only was a female. The four boys exhibited signs of hemorrhagic diathesis (profuse persistent bleeding from insignificant wounds; frequent copious bleeding from the nose) in infancy. At the time of the doctor's visit they were bleeding from the nose, and showed several blood tumors under the skin of their limbs, and numerous petechial spots. Two of the patients belonged to the poorest class; but one was the child of very wealthy people, and two lived in fair circumstances. In regard to treatment, the fluid extract of ergot in moderate doses often repeated, and combined with tincture of digitalis, seemed to control the attack of hemorrhage, to lengthen the period of intermission, and to lessen the severity of subsequent attacks. In his remarks, Dr. D. touched upon the various theories which have been advanced, but none of which offers a satisfactory explanation of the peculiar features of purpura hemorrhagica.

In the discussion following the report of Dr. Davis, Dr. HOLMES related a remarkable case of hemorrhagic diathesis, which lately came under his observation. About one year ago, an apparently healthy old man, with senile cataract in both eyes, was admitted to the Eye and Ear Infirmary. Dr. H. performed the preliminary operation of iridectomy upon both eyes, and though there was more bleeding than usually, he did not suspect there was anything wrong. The eyes recovered quickly, and the patient left the institution after two weeks. In November, he returned for the extraction of the cataract. The operation went off without any accident, except a rather profuse bleeding from the conjunctival flap. The eye progressed favorably until the third day after the operation. On that day, without any apparent cause, the blood began oozing out of the wound, and continued flowing for nine days in spite of pressure bandage, and the internal use of ergot. The patient then stated that on several occasions he nearly bled to death from slight wounds, and the bleeding always began on the third day.

